# Optimization of the Embryo Transfer Technique in the Korean Native Cattle: Effects of Key Influencing Factors

**DOI:** 10.3390/ani16010125

**Published:** 2026-01-01

**Authors:** Seungki Jung, Heejae Yang, Yeonsub Jung, Minki Lee, Hyeonseok Sul, Yeon-Gil Jung, Joohyeong Lee, Sang-Hwan Hyun

**Affiliations:** 1 Veterinary Medical Center, Laboratory of Veterinary Embryology and Biotechnology (VETEMBIO), College of Veterinary Medicine, Chungbuk National University, Cheongju 28644, Republic of Korea; jungseungki2021@gmail.com; 2 ET Biotech Co., Ltd., Jangsu 55609, Republic of Korea; a012cool@naver.com (Y.J.); solhyonsok@hanmail.net (H.S.); jygil1999@hanmail.net (Y.-G.J.); 3 Cocoon Inc., Cheongju 28161, Republic of Korea; hrbidhm0419@gmail.com (H.Y.); lmg950622@gmail.com (M.L.); 4 Department of Companion Animal Industry, Semyung University, Jecheon 27136, Republic of Korea; 5 Institute of Stem Cell & Regenerative Medicine (ISCRM), Chungbuk National University, Cheongju 28644, Republic of Korea; 6 Vet-ICT Convergence Education and Research Center (VICERC), Chungbuk National University, Cheongju 28644, Republic of Korea; 7 Chungbuk National University Hospital, Cheongju 28644, Republic of Korea

**Keywords:** bovine, embryo transfer, metabolic profile test, ovum pickup, pregnancy rate

## Abstract

The success rate of embryo transfer (ET) is influenced by various factors, including embryo quality, environmental conditions, and recipient cows. This study examines the impact of ET on embryo success rates using embryos produced using the ovum pickup method, with fresh and frozen embryos generated on day 7 after in vitro fertilization and subsequently implanted into recipient cows. The factors that contributed to the success rate of ET were investigated, with a focus on embryo type, parity, and farm management. Furthermore, metabolic profile tests were conducted to determine the factors underlying the observed differences in pregnancy rates across the farms. The analysis revealed that pregnancy rates did not significantly differ according to embryo type or parity. However, significant differences (*p* < 0.05) were observed in the glucose, cholesterol, non-esterified fatty acid, total protein, globulin, albumin/globulin, and aspartate aminotransferase levels of the recipients. Furthermore, a comparison of farms with high and low pregnancy rates revealed significant differences in the glucose, cholesterol, total protein, albumin, globulin, and albumin/globulin levels (*p* < 0.05). Differences between farms were associated with the feed management strategy, which highlights the significance of optimal recipient cow management.

## Simple Summary

This study investigated the factors that contribute to the success rate of embryo transfer, with a particular focus on the influence of embryo type, parity, and farm management. Furthermore, a metabolic profile test was conducted to determine the factors underlying the observed differences in conception rates across farms. The analysis revealed no statistically significant differences in the conception rate according to embryo type or parity. However, differences in the conception rate were observed among the farms, with significant differences observed in the glucose, cholesterol, non-esterified fatty acid, total protein, globulin, albumin/globulin, and aspartate aminotransferase levels of the recipients. The findings revealed that differences in conception rate were not observed based on the type of embryo or parity, although differences were observed between farms. This outcome can be attributed to the effect of feed management strategies employed between the farms, which highlights the significance of optimal management of recipient cows. Furthermore, substantial disparities between farms were observed based on the metabolic profile test analysis. The findings show that metabolic profile tests can provide a standard numerical criterion for the prospective management of recipient cows.

## 1. Introduction

Embryo transfer (ET) produces a greater number of genetically superior bovine offspring than other breeding techniques by maximizing the genetic potential. Moreover, compared to artificial insemination (AI), ET can produce a greater number of offspring within a relatively brief period, thereby facilitating genetic selection. This process is now being accelerated by ovum pickup (OPU), a method that involves repeatedly obtaining oocytes from live cows to produce embryos. OPU enables more efficient embryo and offspring production per donor within a shorter period of time compared to multiple ovulation embryo transfer (MOET). Moreover, its dependence on the reproductive status of the donor cow is less than that of MOET [1]. Slaughterhouse-derived in vitro embryo production entails a singular collection of donor oocytes, whereas OPU facilitates repeated oocyte retrieval from a single donor and the subsequent production of in vitro embryos. According to a report by the International Embryo Technology Society (IETS), the number of in vitro ETs has been increasing since 2004, with 1,179,027 ovum pickup (OPU) in vitro fertilization (IVF) ETs performed in 2022 [2]. Although the number of in vivo-derived (IVD) ETs has been declining since 2016, the transfer of IVF-derived embryos has increased due to the commercialization of OPU. Meanwhile, although there is no officially reported data in Korea, the utilization of in vitro embryo production has increased significantly compared to in vivo embryo production, and the utilization of OPU-IVF in the field has further increased since 2018. However, as is the case in other countries, Korea also exhibits low conception rates for ET in some farms, resulting in lower reproduction by ET compared to AI.

Numerous studies have documented substantial discrepancies in pregnancy rates via ET [3,4,5] based on the quality and stage of the embryo, time of completion, and location of the ET within the uterus [6]. As demonstrated in earlier research, IVD embryos have been shown to have a higher pregnancy rate than IVF embryos [1], which may be attributable to inherent variations between IVD and IVF [7,8,9]. Furthermore, the pregnancy rate of each farm has been reported to be contingent upon the management practices applied to the recipient cow [10]. The management of recipient cows is influenced by a number of factors, including lactation [11], body condition score [12], parity [13], season [14], and corpus luteum quality [10]. These factors are pivotal in achieving a high pregnancy rate and must be further investigated to improve IVF outcomes.

As reported by Hansen et al. [15], the pregnancy rate during the summer season is significantly reduced by AI compared to that in the winter period. However, the pregnancy rate facilitated by ET is less affected by seasonal factors. This finding indicates that culturing oocytes in an incubator may serve as a strategy to mitigate the deleterious effects of heat stress, with evidence suggesting that ET is more efficacious than AI during summer. Furthermore, the deterioration of embryo quality due to oxidative stress and cell death during the freezing process can also contribute to decreases in the pregnancy rate [16,17]. Reduced embryo quality is a factor that affects ET and leads to differences in pregnancy rates between fresh and frozen ET. To address this challenge, freezing solutions have been developed and refined [18,19]. Effective feed management is another important factor in the success of ET [20]. Furthermore, the heterogeneity in feed management practices across farms leads to considerable variation in ET outcomes. One solution to this issue is to create a consistent indicator for farm feed.

Metabolic profile tests (MPTs) represent objective indicators that can be used to evaluate farm feed management. These tests are performed to ascertain the metabolic state of an individual and evaluate their health conditions. The indicators that can be observed through MPTs include glucose, cholesterol, non-esterified fatty acid (NEFA), blood urea nitrogen (BUN), albumin, globulin, gamma-glutamyl transferase (GGT), and aspartate aminotransferase (AST) [21,22]. The system enables the monitoring of farm feed management, thereby facilitating the comprehensive and standardized management of cattle. Furthermore, MPTs are closely associated with the management of cattle breeding. In dairy cows, milk production and breeding are closely related, and breeding efficiency can be enhanced through the use of MPTs [23]. Reproductive capacity must be enhanced through optimal management strategies, not only in dairy cows but also in beef cattle. In particular, ET imposes a considerable financial burden, thus necessitating the development of an indicator capable of enhancing the pregnancy rate through MPTs.

This study examined the outcomes of commercial ET and MPTs conducted from 2022 to 2024. The pregnancy rates following ET at each farm were evaluated using embryos produced under a consistent protocol at a single laboratory and by a single ET technician. Additionally, the metabolic status of the farms was analyzed via MPTs, with a focus on the differences resulting from group feeding management and its effect on ET.

## 2. Materials and Methods

### 2.1. Location and Animals

OPU was conducted in Boeun-gun, Republic of Korea. The ET experiment was conducted at 21 farms located in various parts of Boeun-gun, and the experimental subjects were recipient cows managed at these farms.

### 2.2. Experimental Design

#### 2.2.1. Effects of ET on Pregnancy Rates

ET was performed on 21 farms between June 2022 and November 2024. The recipient cows were of Korean origin and exhibited parity ranging from 1 to ≥3. Prior to ET, recipient cows were subjected to a rectal examination to evaluate the presence of reproductive disorders, and ET was performed exclusively on cows exhibiting normal estrous cycles, either spontaneously or after estrus synchronization. (i) Pregnancy rates after ET were determined in 2022, 2023, and 2024. (ii) The pregnancy rates of both fresh and frozen embryos used in the ET procedure were investigated. (iii) ET results were further classified according to the parity of the recipient cows. (iv) A total of 21 farms participated in this transplant project, and 17 of them participated in the MPT analysis. The analysis was not conducted on the remaining four farms due to their decision not to participate. Consequently, the results of the MPT analysis using blood samples from these 17 farms were collected.

#### 2.2.2. Results of Pregnancy Rate After ET and MPT Analysis

Prior to ET, blood samples were collected from the cows and subjected to MPT analysis. (i) Farms exhibiting differences in pregnancy rates after ET were selected, and these differences were substantiated through MPT analysis. (ii) The correlation between each MPT result and pregnancy rate was investigated. (iii) Differences in the MPT analysis and conception rate results between farms were evaluated. Consequently, farms exhibiting below-average pregnancy rates with an average conception rate of 41.1% were designated as the Low group, while farms demonstrating above-average pregnancy rates were classified as the High group. The MPT outcomes of these groups were then thoroughly analyzed. The MPT analysis results for (ii) and (iii) included data from 17 farms that performed MPT. Blood samples were collected from a subset of recipient cows that underwent embryo transfer, irrespective of pregnancy status, and from other reproductively active cows on the same farms for metabolic profile testing. This approach was adopted to evaluate the collective metabolic status of the herd environment as opposed to the metabolic status based on pregnancy status or individual recipient cows.

### 2.3. Embryo Production

#### 2.3.1. OPU

The donor cows utilized in the OPU were of the native Korean cattle breed. Additionally, the subjects were raised on a single farm, and the OPU was performed by a single technician. OPU was conducted in accordance with routine commercial field practices, without a standardized experimental protocol. Prior to undergoing the OPU procedure, the donor was administered epidural anesthesia via the injection of 5 mL of lidocaine into the sacrococcygeal space. Following the meticulous cleansing of the vulva and perineum to ensure the removal of fecal material, a follicle aspiration guide (WTA^®^, Cravinhos SP, Brazil) was inserted in conjunction with a probe (4Vet Slim, Draminski, Olsztyn, Poland) with a 2–8 MHz convex array transducer to the floor of the vaginal sac. Follicles were then aspirated using a 20 G disposable needle system (WTA^®^) connected to a silicone tubing line and equipped with a 50 mL tube heater and a vacuum pump. Follicles with a diameter greater than 2 mm, as visualized by ultrasound, were subjected to aspiration, and the follicular fluid containing the oocytes was transferred to a 50 mL tube containing OPU washing medium, which consisted of 1% fetal bovine serum (FBS; Access Biologicals, Vista, CA, USA) and 10 IU/mL heparin (JW Pharmaceutical, Gwacheon-si, Gyeonggi-do, Republic of Korea) in Hartmann’s solution (JW Pharmaceutical). The samples were then stored at 38 °C until oocyte retrieval.

#### 2.3.2. Oocyte Retrieval and In Vitro Maturation

The retrieved cumulus cell–oocyte complexes (COCs) were transferred to an embryo collection filter (Cell Collector; Fujihira Industry, Tokyo, Japan) and then washed with the same solution used for the recovery process. Thereafter, COCs that possessed a minimum of one layer of cumulus cells and homogeneous cytoplasm were meticulously sorted from the sediment. In vitro maturation (IVM) was performed according to the method described by Jung et al. [19]. The COCs were washed three times with OCM (IFP9611; Research Institute for the Functional Peptides, Yamagata, Japan). Thereafter, the selected COCs were washed three times with HP-M199 (IFP971; Research Institute for the Functional Peptides). The maturation medium was then supplemented with 0.5 μg/mL follicle-stimulating hormone (Folltropin V; Bioniche Animal Health, Belleville, ON, Canada) and 5% FBS and used as the IVM medium. COCs were randomly cultured in 5-well dishes with 50 COCs per well (1 mL IVM medium per well) at 38.9 °C, 5% CO_2_, and 100% humidity for 22 h.

#### 2.3.3. IVF and In Vitro Culture

IVF was performed as previously described by Jung et al. [19]. Semen from Korean cattle was cryopreserved at −196 °C in 0.5 mL straws until required. The straws were thawed in a 38 °C water bath for 40 s, after which the thawed sperm were transferred into a 15 mL conical tube containing 1 mL of SPF45 and 1 mL of 80 SpermFilter^®^ (Gynotec, Malden, The Netherlands). The tubes were then centrifuged at 600× *g* for 15 min. The precipitate was transferred to a new centrifuge tube containing 4 mL IVF100 (IFP9630; Research Institute for the Functional Peptides) and subjected to further centrifugation at 600× *g* for 10 min. For IVF, approximately 10–15 expanded oocytes surrounded by cumulus cells were placed in 50 μL of IVF medium. The precipitate was then diluted with IVF medium to obtain a concentration of 1 × 10^7^ sperm/mL. Subsequently, the adjusted IVF medium was added to the drop containing the oocytes, resulting in a final concentration of 2 × 10^6^ sperm/mL. The IVF medium was then cultured for 6 h at 38.9 °C, 5% CO_2_, and 100% humidity. The day of fertilization was labeled as day 0. At the end of IVF, the presumptive zygote was removed from the cumulus cells and sperm by pipetting. For in vitro culture (IVC), 10–15 presumptive zygotes were placed in a 50 μL drop of potassium simplex optimized medium and incubated at 38.9 °C under 5% CO_2_, 5% O_2_, and 100% humidity until the blastocyst stage.

#### 2.3.4. Slow Freezing and Thawing

All embryo transfers were performed using good-quality blastocysts according to IETS guidelines [24]. Blastocysts were washed three times in freezing medium containing ethylene glycol and then equilibrated in the same medium for 15 min at 20–25 °C. During freezing medium exposure, a single blastocyst was placed into each 0.25 mL straw (IMV Technologies, L’Aigle, France). Following exposure, the straws were placed in a freezer (FREEZE CONTROL^®^; Cryologic, Victoria, Australia) at −6 °C. After 2 min, seeding was induced using a pair of tweezers to prevent supercooling. After another 10 min, the straws were frozen to −30 °C at a rate of −0.3 °C per min. Subsequently, the straws were stored in liquid nitrogen.

#### 2.3.5. Synchronization of Estrus and ET

An estrus synchronization protocol was implemented to facilitate ET at a predetermined time. On day 0, Cue-Mate^®^ (Vetoquinol Australia, Hamilton, QLD, Australia) was inserted intravaginally in all recipients. On day 7, Cue-Mate^®^ was removed, and 5 mL of Lutalyse^®^ (Zoetis Inc., Kalamazoo, MI, USA) was injected intramuscularly. On day 9, 3 mL of Gonadon (Dong Bang Co., Ltd., Anseong-si, Gyeonggi-do, Republic of Korea) was injected intramuscularly. On day 16, the presence of the corpus luteum was confirmed by ultrasound examination, and ET was performed on recipient cows whose corpus luteum size was 1 cm or larger. Frozen embryos were transferred to the uterus of the recipient cows after thawing. The thawing process was conducted by placing frozen straws containing blastocysts in air for a duration of 10 s, followed by submersion in water at a temperature of 35 °C for a period of 20 s. The recovered blastocysts were washed three times with Dulbecco’s phosphate-buffered saline containing 10% FBS, diluted in IVC medium for 10 min, and cultured in the same medium at 38.9 °C, 5% CO_2_, 5% O_2_, and 100% humidity. The recovery of blastocysts was monitored at 1 h intervals, and embryos were transferred as required. Pregnancy was diagnosed using ultrasound imaging at least 40 days after ET. The ET procedure was performed by a single technician.

### 2.4. Blood Collection

For the MPT test, the cows were safely restrained, and blood was collected from the neck area by a veterinarian. A 5 mL syringe with a 19 G needle was used to collect blood samples, with approximately 5 mL of blood obtained from each subject. After collection, blood samples were transferred to a 5 mL serum separator tube at the point of collection. The tubes were then stored in a refrigerator. After collection, the blood samples were centrifuged (Sorvall Legend ST16R; Thermo Fisher Scientific, Waltham, MA, USA) at 3000 RPM for 6 min. This procedure was performed to facilitate serum separation.

### 2.5. MPT Analysis

In the MPT analysis, 4 out of 21 farms were excluded, while data from the 17 remaining farms were ultimately included in the analysis. The MPT results were derived from the data provided by these 17 farms, with samples for which values were not properly derived being excluded during the analysis. Consequently, 148 samples were used to assess the glucose, cholesterol, NEFA, total protein, albumin, globulin, and BUN levels, whereas 140 and 134 samples were used to estimate the AST and GGT levels, respectively, excluding errors. The embryo transfer project was conducted commercially, and MPT analysis was only conducted in 2023, not in 2022 or 2024. The MPT analysis was conducted in a commercial diagnostic laboratory using a Selectra E biochemical analyzer (Vital Scientific, Spankeren, The Netherlands). Despite the lack of detailed validation parameters such as precision and LOD, this analyzer is widely used in routine veterinary diagnostics and recognized for its reliability. This study used field data collected from 17 farms under consistent embryo production and ET protocols.

### 2.6. Statistical Analysis

Pregnancy outcome data, including the year, embryo type, parity, and pregnancy rates across the farms, were analyzed using a generalized linear mixed model in SPSS (version 30.0; IBM Corp., Armonk, NY, USA). For Table 1, year, embryo type, and parity were included as fixed effects, and farm was included as a random effect. For Table 2, year, embryo type, parity, and farm were included as fixed effects. Statistical comparisons were performed using Bonferroni-adjusted pairwise comparisons based on estimated marginal means. All other analyses were conducted using GraphPad Prism software (version 8.0.1; GraphPad Software, San Diego, CA, USA). MPT data across farms were analyzed using one-way analysis of variance (ANOVA), followed by Tukey’s multiple-comparison test. Pearson’s correlation and simple linear regression analyses were performed to evaluate the relationships between pregnancy rates and MPT variables. Coefficients of determination (R^2^), regression equations, and *p*-values were calculated. Differences in MPT results between Low and High pregnancy rate groups were assessed using independent *t*-tests. Statistical significance was defined as *p* < 0.05.

## 3. Results

### 3.1. Impact of Various Factors on ET

A total of 382 embryos were transferred into recipient cows, and 160 recipient cows achieved pregnancy (41.9%, Table 1). The pregnancy rates for the years 2022, 2023, and 2024 are presented in Table 1. Significant differences were not observed in the pregnancy rate of ET in each year. The study further categorized the transferred embryos into two groups: fresh embryos and frozen embryos. However, the analysis revealed no statistically significant differences between these two groups (Table 1). The parity of recipient cows was divided into 0, 1, 2, and ≥3, and significant differences in pregnancy rates were not observed between each group (Table 1). The pregnancy rates of each of the 17 farms are shown in Table 2. A significant difference was observed in the pregnancy rates of farms A, B, and C compared to farm Q (*p* < 0.05).

### 3.2. Pregnancy Rates After ET Based on the MPT Results

**Figure 1:**
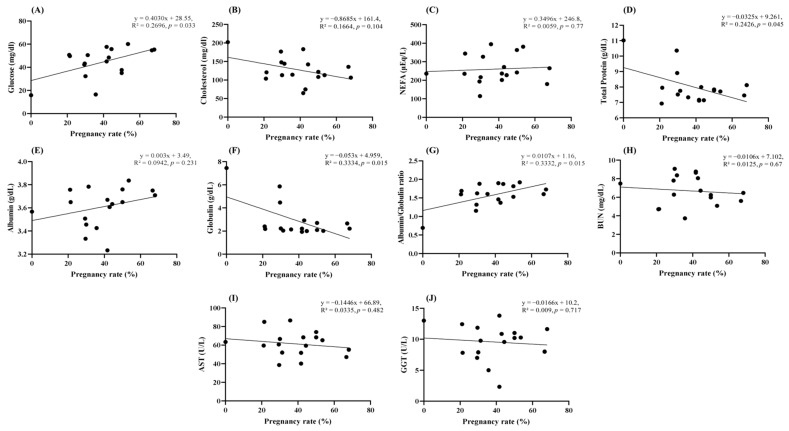
Scatterplot showing the correlation between pregnancy rate and metabolic profile parameters of recipients. The following variables were analyzed: (**A**) glucose (mg/dL), (**B**) cholesterol (mg/dL), (**C**) non-esterified fatty acid (NEFA, µEq/L), (**D**) total protein (g/dL), (**E**) albumin (g/dL), (**F**) globulin (g/dL) (**G**) albumin/globulin ratio (A/G), (**H**) blood urea nitrogen (BUN, mg/dL), (**I**) aspartate aminotransferase (AST, IU/L), and (**J**) gamma-glutamyl transferase (GGT, IU/L). A substantial correlation was identified for glucose (y = 0.4030x + 28.55, R^2^ = 0.2696, *p* = 0.0327), total protein (y = −0.0325x + 9.261, R^2^ = 0.2426, *p* = 0.045), globulin (y = −0.053x + 4.959, R^2^ = 0.3334, *p* = 0.015), and A/G ratio (y = 0.0107x + 1.16, R^2^ = 0.3332, *p* = 0.015).

**Figure 2:**
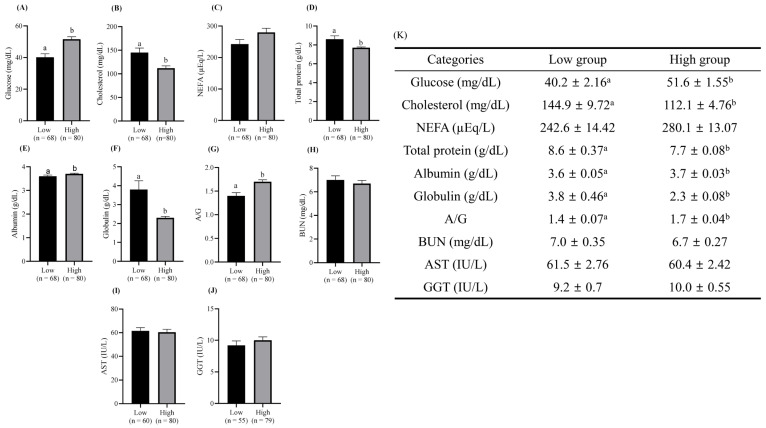
Comparison of metabolic profile parameters between the High and Low groups following embryo transfer. Each bar plot represents the mean ± standard error of the mean of (**A**) glucose (mg/dL), (**B**) cholesterol (mg/dL), (**C**) non-esterified fatty acid (NEFA, µEq/L), (**D**) total protein (g/dL), (**E**) albumin (g/dL), (**F**) globulin (g/dL), (**G**) albumin/globulin ratio (A/G), (**H**) blood urea nitrogen (BUN, mg/dL), (**I**) aspartate aminotransferase (AST, IU/L), and (**J**) gamma-glutamyl transferase (GGT, IU/L) for the High and Low groups. (**K**) A table showing the figures for each category. ^ab^ Indicate statistically significant differences between the two groups (*p* < 0.05).

As demonstrated in Table 2, the results of the MPT analysis were examined for farms that exhibited substantial variations in pregnancy rates. The findings of this analysis are presented in Table 3. A significant difference was observed in glucose, globulin, and albumin/globulin (A/G) levels between farm Q and farms A, B, and C (*p* < 0.05). In contrast, a significant increase in cholesterol was observed in farm Q compared to farms A and C (*p* < 0.05). The NEFA levels were significantly higher in farm C than in farms A, B, and Q (*p* < 0.05). The total protein levels in farms B and C were significantly lower than those in farm Q (*p* < 0.05). A significant difference in AST levels was also observed between farms B and C (*p* < 0.05). As illustrated in Figure 1, the results of each MPT item were correlated with the pregnancy rate after ET. The findings indicated weak but statistically significant correlations between glucose (R^2^ = 0.2696, *p* = 0.033) and A/G (R^2^ = 0.3332, *p* = 0.015) levels and pregnancy rates. Conversely, the total protein (R^2^ = 0.2426, *p* = 0.045) and globulin (R^2^ = 0.3334, *p* = 0.015) levels showed weak but statistically significant negative correlations with the pregnancy rate. As illustrated in Figure 2, the MPT results were divided into Low and High groups based on the pregnancy rate. The Low group exhibited significantly lower glucose, albumin, and A/G levels relative to the High group (*p* < 0.05). Conversely, the Low group exhibited significantly higher levels of cholesterol, total protein, and globulin compared to the High group (*p* < 0.05).

## 4. Discussion

The present experiment was conducted to investigate the effect of ET on pregnancy rates in cattle. Furthermore, we investigated the pregnancy rate by farm and determined the factors underlying these differences in pregnancy rate through MPT analysis. We then assessed the correlation between pregnancy rate and each component of the MPT analysis and differences in the MPT results between the Low and High pregnancy rate groups.

Embryo quality is a pivotal factor in determining the success of ET, with a demonstrable relationship between the quality of the embryo and the subsequent pregnancy rate [25]. This outcome can be attributed to the fact that embryos experience apoptosis and deteriorated quality due to osmotic stress and cell damage caused by ice crystal formation during the freezing process [26]. Furthermore, freezing embryos can increase ROS levels based on the associated decline in mitochondrial function [16]. Our field results demonstrated that the pregnancy rate after ET did not significantly differ between fresh and frozen embryos. In this study, the frozen embryos were not thawed for direct transfer in the field but rather were selected and transferred after thawing and cultured in the laboratory. These outcomes were contrary to those of conventional frozen embryos. The importance of frozen embryos has been emphasized in recent years, and the number of research reports in this area has increased [19,27,28]. Therefore, improvements in this field are required. The parity of cows has a demonstrable impact on pregnancy rates after ET [29,30], with heifers exhibiting a higher pregnancy rate relative to multiparous cows. This is due to the reduced degree of uterine damage or infection and imbalanced uterine environment in heifers relative to multiparous cows. Furthermore, multiparous cows exhibit a higher prevalence of hormonal imbalance and anovulation, which are attributed to metabolic stress and energy deficiency following parturition [31,32]. However, the present study did not confirm a significant difference in the pregnancy rate after ET between parities of 0, 1, 2, and ≥3. This is presumed to be due to variables caused by the feeding management of each farm, as indicated by the comprehensive data collected from 17 farms.

MPTs are used as management indicators for farms and cows [33]. Glucose, NEFA, and cholesterol levels are used as energy-related indicators to reflect the energy status of cows. Total protein is defined as the total protein content in the serum and primarily consists of albumin and globulin. Albumin, a major protein synthesized in the liver, is an indicator of liver function, whereas globulin is an indicator of immune status. Furthermore, BUN is a byproduct of protein metabolism, and its levels are indicative of an individual’s protein status. AST and GGT levels are also used as indicators of liver function. By quantifying the condition of the cows, the MPT results provide feedback on management within the farm. However, the feeding management of the recipient cows raised on each farm differed significantly, which resulted in variations in the pregnancy rate after ET for each farm [34,35]. In this study, a wide range of pregnancy rates was observed, ranging from 0 to 68% across the farms. Because the application of uniform feeding management strategies across all farms is impractical, we hypothesized that these differences in pregnancy rates could be attributed to the feeding regimen. However, given the complexity of analyzing the feeding management of each of the 17 farms, the metabolic status of the breeding cows on each farm was instead analyzed through MPT. The analysis included pregnant cows, non-pregnant cows, and some breeding cows from each farm. While individual MPT analysis results could be derived, this would be confusing, so this analysis focused on the metabolic status of the entire cow herd as a farm group. The MPT analysis of farms A, B, C, and Q showed differences in the glucose, cholesterol, NEFA, total protein, globulin, A/G, and AST levels. Baek et al. [36] reported that when comparing a forage-based feeding management system with a high-energy feed-centered feeding system aimed at improving animal welfare, significant differences were observed in some blood metabolic indicators (MPT items). This finding lends further credence to the hypothesis that discrepancies in feeding management practices across farms can contribute to the observed variation in MPT results. However, given that this study collected data from multiple farms based on retrospective results, it was difficult to describe the feed composition or management systems of each farm in detail. Consequently, the present study centered its interpretation on the potential influence of discrepancies in feeding management practices among farms on the outcomes of the MPT analysis.

In this study, we confirmed the correlation between each MPT result and the pregnancy rate. Although glucose levels and A/G ratios exhibited weak correlations with pregnancy rates, the correlations were statistically significant. This finding is consistent with the conclusions of previous studies [23] in which adequate energy levels were shown to have a positive impact on pregnancy rates. Furthermore, an increase in glucose levels following parturition has been shown to enhance the probability of conception. This finding suggests that glucose plays a critical role in the optimal functioning of the ovary. Conversely, a significant and weak negative correlation was observed between the total protein and globulin levels and the pregnancy rate. Total protein consists of albumin and globulin, and previous reports [37] have indicated that globulin affects the production of embryos in vivo. This finding suggests a potential impact of globulin on reproductive function, with the present study indicating that an increase in globulin levels may have contributed to the observed decrease in pregnancy rates by augmenting the inflammatory index.

The findings of the present study indicate a relationship between the results of the MPT and the success of conception. Therefore, we examined the differences in MPT analysis results between groups based on conception rates. The average pregnancy rate among the 319 recipient cows subjected to ET from 17 farms was 41.1%. After classifying the recipient cows into High and Low pregnancy rate groups based on 41.1%, the differences in metabolic profiles between the two groups were determined. Differences in glucose, cholesterol, total protein, globulin, and A/G levels were observed between the High and Low groups. Glucose is an energy-related indicator that supports the successful pregnancy rate of the High group by providing sufficient energy levels. Sakagami et al. [38] reported that the addition of glucose to embryo culture medium at a designated time period resulted in the enhancement of blastocyst formation. This finding supports the hypothesis that glucose plays an essential role not only in reproduction but also in the development of oocytes and zygotes, which are crucial for reproduction. Mohebbi-Fani et al. [39] reported a negative correlation between glucose and NEFA, suggesting that lower glucose concentrations increase body fat mobilization, leading to hormonal changes and reproductive failure. Takahashi et al. [40] reported that when embryos recovered from Japanese black cows with cholesterol levels of 117.4 mg/dL were transplanted, the pregnancy rate was improved compared to that of embryos recovered from control cows (95.0 mg/dL). In our results, the High group showed a similar value of 112.1 mg/dL, whereas the Low group had an excessively high level of 144.9 mg/dL, suggesting that the pregnancy rate was lowered due to fatty liver, with the resulting energy excess decreasing liver function. Previous studies have reported an association between decreases in protein markers, such as albumin and globulin, along with cholesterol, and postpartum uterine disease, suggesting a potential impact on reproductive function [41]. The inflammatory response of embryos to ET interferes with maternal pregnancy recognition and reduces placental weight, even if the pregnancy is maintained [42]. Furthermore, Rowlands et al. [43] reported that decreased albumin and increased globulin concentrations were also associated with reduced reproductive efficiency. The A/G ratio has been identified as a potential indicator of chronic inflammation. Okawa et al. [37] analyzed A/G levels through blood collection in MOET donor cows and found that cows with low anti-Müllerian hormone (AMH) concentrations exhibited low A/G ratios. Moreover, a negative correlation was identified between the AMH and α_1_-globulin concentrations, suggesting that these cows were likely experiencing an inflammatory state. This inflammatory state has been linked to a decline in the efficiency of embryo production through MOET. The findings of the present study also confirmed that the A/G and globulin concentrations differed between the Low and High groups, suggesting that recipient cows in the Low group had higher inflammation levels than those in the High group. Furthermore, the findings indicated that differences in total protein, including albumin, globulin, and A/G, ultimately demonstrated that changes in the level of inflammation in cows affected conception rates after ET.

Previous studies have assessed reproductive performance using only a limited number of MPT parameters. However, the present study analyzed a total of ten parameters, suggesting that changes in a single metabolic marker may not directly affect conception rates. Rather, the overall metabolic status, including multiple parameters, may interact in a complex manner. Consequently, rather than emphasizing individual parameters, meticulous monitoring and adjustment of overall metabolic balance are imperative. Conception rates can also be influenced by factors such as overall farm management, stress, individual health, and genetic factors. Therefore, future studies should adopt a more comprehensive approach that considers these factors in addition to metabolic status. Moreover, given the retrospective and commercial nature of this field study, subsequent investigations should control for embryo-related variables, particularly by using embryos derived from the same donor cows, and further examine the effects of embryo characteristics on reproductive outcomes.

## 5. Conclusions

In this study, we investigated the pregnancy rate after ET based on embryos derived from a uniform production process performed by the same technician in the same region between 2022 and 2024. Thus, differences in pregnancy rate were not caused by the type of embryo or parity. However, differences were observed between farms. These outcomes are ascribed to the metabolic consequences of feed management between farms, as evidenced by the MPT analysis in the study. This underscores the importance of optimal management of recipient cows. Furthermore, despite the utilization of 10 MPT items in the study, the MPT is better suited for diagnostic purposes, assessing the metabolic status of cows prior to ET, rather than as a predictive tool for each individual item. The results of this study demonstrated the MPT analysis results of farms with high pregnancy rates, and it is anticipated that when accumulated results are generated in the future, they will be utilized as management indicators for recipient cows to enhance conception rates.

## Figures and Tables

**Table 1:** Pregnancy rates after embryo transfer according to various factors.

Groups	No. of Embryo Transfer	No. of Pregnancy	Pregnancy Rate (%)
Year			
2022	75	26	34.7
2023	248	102	41.1
2024	59	32	54.2
Embryo type			
Fresh	281	116	41.3
Frozen	101	44	43.6
Parity			
0	28	12	42.9
1	99	42	42.4
2	106	36	34.0
≥3	149	70	47.0
Total	382	160	41.9

**Table 2:** Pregnancy rate after embryo transfer between 17 farms.

Farms	No. of Embryo Transfer	No. of Pregnancy	Pregnancy Rate (%)
A	25	17	68.0 ^a^
B	21	14	66.7 ^a^
C	43	23	53.5 ^a^
D	14	7	50.0
E	6	3	50.0
F	27	12	44.4
G	28	12	42.9 *
H	12	5	41.7
I	12	5	41.7
J	14	5	35.7
K	16	5	31.3
L	10	3	30.0
M	27	8	29.6
N	17	5	29.4
O	14	3	21.4
P	19	4	21.1
Q	14	0	0.0 ^b^*
Total	319	131	41.1

^ab^ Values in the same column with different superscript letters are significantly different (*p* < 0.05). * *p* = 0.073.

**Table 3:** Metabolic profile test results for each farm with different pregnancy rates.

Farms	No. of Blood Samples Collected	Glucose (mg/dL)	Cholesterol (mg/dL)	NEFA (µEq/L) ^1^	Total Protein (g/dL)	Albumin (g/dL)	Globulin (g/dL)	A/G ^2^	BUN (mg/dL) ^3^	AST (IU/L) ^4^	GGT (IU/L) ^5^
A	11	55.3 ± 3.0 ^a^	106.4 ± 7.5 ^a^	264.9 ± 33.3 ^a^	8.1 ± 0.3 ^ab^	3.7 ± 0.1	2.2 ± 0.1 ^a^	1.7 ± 0.1 ^a^	6.5 ± 0.6	55.2 ± 4.4 ^ab^	11.6± 1.3
B	8	54.6 ± 3.7 ^a^	135.5 ± 8.2 ^ab^	179.4 ± 27.1 ^a^	7.5 ± 0.4 ^a^	3.8 ± 0.1	2.7 ± 0.4 ^a^	1.6 ± 0.2 ^a^	5.6 ± 1.1	47.3 ± 4.3 ^a^	8.0 ± 1.1
C	11	60.1 ± 3.8 ^a^	113.3 ± 11.7 ^a^	381.0 ± 15.9 ^b^	7.7 ± 0.2 ^a^	3.8 ± 0.1	2.0 ± 0.0 ^a^	1.9 ± 0.1 ^a^	5.1 ± 0.6	65.2 ± 4.4 ^b^	10.3 ± 1.6
Q	6	15.8 ± 6.2 ^b^	202.2 ± 56.4 ^b^	235.8 ± 53.5 ^a^	11.0 ± 2.1 ^b^	3.6 ± 0.3	7.5 ± 2.3 ^b^	0.7 ± 0.2 ^b^	7.5 ± 1.0	63.4 ± 6.0 ^ab^	13.0 ± 4.0

^1^ Non-esterified fatty acid. ^2^ Albumin/globulin. ^3^ Blood urea nitrogen. ^4^ Aspartate aminotransferase. ^5^ Gamma-glutamyl transferase. Data are presented as mean ± standard error. ^ab^ Values in the same column with different superscript letters are significantly different (*p* < 0.05).

## Data Availability

Although the data are not publicly available, they can be obtained from the corresponding author upon reasonable request.

## Abbreviations

The following abbreviations are used in this manuscript:

A/G	Albumin/globulin
AI	Artificial insemination
AMH	Anti-Müllerian hormone
ANOVA	Analysis of variance
AST	Aspartate aminotransferase
BUN	Blood urea nitrogen
COC	Cumulus–oocyte complex
ET	Embryo transfer
FBS	Fetal bovine serum
GGT	gamma-glutamyl transferase
IVC	In vitro culture
IVD	In vivo derived
IVF	In vitro fertilization
IVM	In vitro maturation
MOET	Multiple ovulation embryo transfer
MPTs	Metabolic profile tests
NEFA	Non-esterified fatty acid
OPU	Ovum pickup

## References

[1] Pontes J.H., Nonato-Junior I., Sanches B.V., Ereno-Junior J.C., Uvo S., Barreiros T.R., Oliveira J.A., Hasler J.F., Seneda M.M. (2009). Comparison of embryo yield and pregnancy rate between in vivo and in vitro methods in the same Nelore (*Bos indicus*) donor cows. Theriogenology.

[2] Viana J. (2022). 2021 statistics of embryo production and transfer in domestic farm animals. Embryo Technol. Newsl..

[3] Pugliesi G., Guimaraes da Silva A., Viana J.H.M., Siqueira L.G.B. (2023). Review: Current status of corpus luteum assessment by Doppler ultrasonography to diagnose non-pregnancy and select embryo recipients in cattle. Animal.

[4] Morotti F., Dos Santos G.M.G., Silva-Santos K.C., Dias J.H.A., Seneda M.M. (2025). Strategic use of estrus intensity to combine timed artificial insemination and embryo transfer in large-scale cattle reproduction programs. Theriogenology.

[5] Karasahin T., Alkan H., Satilmis F., Dursun S., Erdem H. (2021). Effect of flunixin meglumine treatment during and after embryo transfer on the pregnancy rate in cattle. Reprod. Domest. Anim..

[6] Alkan H., Karasahin T., Dursun S., Satilmis F., Erdem H., Guler M. (2020). Evaluation of the factors that affect the pregnancy rates during embryo transfer in beef heifers. Reprod. Domest. Anim..

[7] Khurana N.K., Niemann H. (2000). Energy metabolism in preimplantation bovine embryos derived in vitro or in vivo. Biol. Reprod..

[8] Corcoran D., Rizos D., Fair T., Evans A.C., Lonergan P. (2007). Temporal expression of transcripts related to embryo quality in bovine embryos cultured from the two-cell to blastocyst stage in vitro or in vivo. Mol. Reprod. Dev..

[9] Noguchi T., Aizawa T., Munakata Y., Iwata H. (2020). Comparison of gene expression and mitochondria number between bovine blastocysts obtained in vitro and in vivo. J. Reprod. Dev..

[10] Siqueira L.G., Torres C.A., Souza E.D., Monteiro P.L., Arashiro E.K., Camargo L.S., Fernandes C.A., Viana J.H. (2009). Pregnancy rates and corpus luteum-related factors affecting pregnancy establishment in bovine recipients synchronized for fixed-time embryo transfer. Theriogenology.

[11] Lopez H., Satter L.D., Wiltbank M.C. (2004). Relationship between level of milk production and estrous behavior of lactating dairy cows. Anim. Reprod. Sci..

[12] Bonacker R.C., Gray K.R., Breiner C.A., Anderson J.M., Patterson D.J., Spinka C.M., Thomas J.M. (2020). Comparison of the 7 & 7 Synch protocol and the 7-day CO-Synch + CIDR protocol among recipient beef cows in an embryo transfer program. Theriogenology.

[13] Ferraz P.A., Burnley C., Karanja J., Viera-Neto A., Santos J.E., Chebel R.C., Galvao K.N. (2016). Factors affecting the success of a large embryo transfer program in Holstein cattle in a commercial herd in the southeast region of the United States. Theriogenology.

[14] Nishisozu T., Singh J., Abe A., Okamura K., Dochi O. (2023). Effects of the temperature-humidity index on conception rates in Holstein heifers and cows receiving in vitro-produced Japanese Black cattle embryos. J. Reprod. Dev..

[15] Hansen P.J., Arechiga C.F. (1999). Strategies for managing reproduction in the heat-stressed dairy cow. J. Anim. Sci..

[16] Lopez-Damian E.P., Jimenez-Medina J.A., Alarcon M.A., Lammoglia M.A., Hernandez A., Galina C.S., Fiordelisio T. (2020). Cryopreservation induces higher oxidative stress levels in Bos indicus embryos compared with Bos taurus. Theriogenology.

[17] Min S.H., Kim J.W., Lee Y.H., Park S.Y., Jeong P.S., Yeon J.Y., Park H., Chang K.T., Koo D.B. (2014). Forced collapse of the blastocoel cavity improves developmental potential in cryopreserved bovine blastocysts by slow-rate freezing and vitrification. Reprod. Domest. Anim..

[18] Jung S., Sul H., Oh D., Jung Y.G., Lee J., Hyun S.H. (2024). Slow freezing cryopreservation of Korean bovine blastocysts with an additional sucrose pre-equilibration step. Front. Vet. Sci..

[19] Jung S., Jung Y., Sul H., Jung Y.G., Ham J., Oh D., Lee J., Hyun S.H. (2025). L-proline supplementation in the freezing medium enhances the viability and quality of bovine blastocysts after slow freezing and thawing. Theriogenology.

[20] Fontes P.L.P., Oosthuizen N., Ciriaco F.M., Sanford C.D., Canal L.B., Cooke R.F., Pohler K.G., Henry D.D., Mercadante V.R.G., Ealy A.D. (2021). Effects of nutrient restriction on the metabolic profile of *Bos indicus*-influenced and *B. taurus* suckled beef cows. Animal.

[21] Kida K. (2002). Use of every ten-day criteria for metabolic profile test after calving and dry off in dairy herds. J. Vet. Med. Sci..

[22] Takasu M., Yayota M., Nakano M., Nishii N., Ohba Y., Okada K., Maeda S., Miyazawa K., Kitagawa H. (2005). Results of metabolic profile test in Japanese black cattle with growth retardation. J. Vet. Med. Sci..

[23] Garverick H.A., Harris M.N., Vogel-Bluel R., Sampson J.D., Bader J., Lamberson W.R., Spain J.N., Lucy M.C., Youngquist R.S. (2013). Concentrations of nonesterified fatty acids and glucose in blood of periparturient dairy cows are indicative of pregnancy success at first insemination. J. Dairy Sci..

[24] Bo G., Mapletoft R. (2013). Evaluation and classification of bovine embryos. Anim. Reprod..

[25] Erdem H., Karasahin T., Alkan H., Dursun S., Satilmis F., Guler M. (2020). Effect of embryo quality and developmental stages on pregnancy rate during fresh embryo transfer in beef heifers. Trop. Anim. Health Prod..

[26] Dochi O. (2019). Direct transfer of frozen-thawed bovine embryos and its application in cattle reproduction management. J. Reprod. Dev..

[27] Carrascal-Triana E.L., Zolini A.M., de King A.R., Penitente-Filho J.M., Hansen P.J., Torres C.A.A., Block J. (2022). Effect of addition of ascorbate, dithiothreitol or a caspase-3 inhibitor to cryopreservation medium on post-thaw survival of bovine embryos produced in vitro. Reprod. Domest. Anim..

[28] Ishii T., Mori-Kobayashi K., Nakamura S., Ohkura S., Matsuyama S. (2024). Carnosine supplementation in cryopreservation solution improved frozen-thawed bovine embryo viability. J. Reprod. Dev..

[29] Dochi O., Yamamoto Y., Saga H., Yoshiba N., Kano N., Maeda J., Miyata K., Yamauchi A., Tominaga K., Oda Y. (1998). Direct transfer of bovine embryos frozen-thawed in the presence of propylene glycol or ethylene glycol under on-farm conditions in an integrated embryo transfer program. Theriogenology.

[30] Lee J., Lee S., Ryu G., Kim D., Baek H.U., Kim J., Lee K., Kim S., Kim S., Dang C.G. (2024). A retrospective analysis of conception per embryo transfer in dairy cattle in South Korea. Theriogenology.

[31] Dimmick M.A., Gimenez T., Spitzer J.C. (1991). Ovarian endocrine activity and development of ovarian follicles during the postpartum interval in beef cows. Anim. Reprod. Sci..

[32] Meikle A., Kulcsar M., Chilliard Y., Febel H., Delavaud C., Cavestany D., Chilibroste P. (2004). Effects of parity and body condition at parturition on endocrine and reproductive parameters of the cow. Reproduction.

[33] Kida K. (2002). The metabolic profile test: Its practicability in assessing feeding management and periparturient diseases in high yielding commercial dairy herds. J. Vet. Med. Sci..

[34] Vasconcelos J.L., Jardina D.T., Sa Filho O.G., Aragon F.L., Veras M.B. (2011). Comparison of progesterone-based protocols with gonadotropin-releasing hormone or estradiol benzoate for timed artificial insemination or embryo transfer in lactating dairy cows. Theriogenology.

[35] Kasimanickam R.K., Hall J.B., Estill C.T., Kastelic J.P., Joseph C., Abdel Aziz R.L., Nak D. (2018). Flunixin meglumine improves pregnancy rate in embryo recipient beef cows with an excitable temperament. Theriogenology.

[36] Baek D.J., Kwon H.C., Mun A.L., Lim J.R., Park S.W., Han J.S. (2022). A comparative analysis of rumen pH, milk production characteristics, and blood metabolites of Holstein cattle fed different forage levels for the establishment of objective indicators of the animal welfare certification standard. Anim. Biosci..

[37] Okawa H., Yukiyama N., Yamato O., Goto A., Widodo O.S., Fushimi Y., Takagi M. (2025). Factors influencing in vivo embryo production in Japanese Black donors: The role of anti-Mullerian hormone and inflammation parameters. J. Reprod. Dev..

[38] Sakagami N., Nishino O., Adachi S., Umeki H., Uchiyama H., Ichikawa K., Takeshita K., Kaneko E., Akiyama K., Kobayashi S. (2014). Improvement of preimplantation development of in vitro-fertilized bovine zygotes by glucose supplementation to a chemically defined medium. J. Vet. Med. Sci..

[39] Mohebbi-Fani M., Omidi A., Mirzaei A., Nazifi S., Nowroozi K. (2019). A field study on glucose, non-esterified fatty acids, beta-hydroxybutyrate and thyroid hormones in dairy cows during the breeding period in Fars province, Iran. Iran. J. Vet. Res..

[40] Takahashi M., Sawada K., Kawate N., Inaba T., Tamada H. (2013). Improvement of superovulatory response and pregnancy rate after transfer of embryos recovered from Japanese Black cows fed rumen bypass polyunsaturated fatty acids. J. Vet. Med. Sci..

[41] Thongrueang N., Yang S.F., Ke G.M., Hsu H.Y., Lee H.H. (2023). Albumin and other metabolic parameters as potential indicators of purulent vaginal discharge in dairy cows during the transition period. J. Vet. Med. Sci..

[42] Chastant S., Saint-Dizier M. (2019). Inflammation: Friend or foe of bovine reproduction?. Anim. Reprod..

[43] Rowlands G.J., Little W., Kitchenham B.A. (1977). Relationships between blood composition and fertility in dairy cows—A field study. J. Dairy Res..

